# An Investigation into PVA Fiber Modified with SiO_2_ for Improving Mechanical Properties of Oil-Well Cements

**DOI:** 10.3390/ma17112581

**Published:** 2024-05-27

**Authors:** Zhiqiang Wu, Zehua Chen, Jingping Liu, Chengwen Wang

**Affiliations:** 1 CNOOC Research Institute Co., Ltd., Beijing 100028, China; wuzhq2@cnooc.com.cn; 2 Key Laboratory of Unconventional Oil & Gas Development of Ministry of Education, College of Petroleum Engineering, China University of Petroleum (East China), Qingdao 266580, China; liujingping20@126.com

**Keywords:** PVA fiber enhanced cement, properties, characterization, mechanism

## Abstract

In this paper, we conduct a comprehensive investigation into PVA fiber modified with SiO_2_ to improve the mechanical properties of oil-well cements. Specifically, SiO_2_ was coated onto the surface of polyvinyl alcohol fiber (PVAF) as its silicon source via a sol-gel process by using tetraethyl orthosilicate (TEOS), while hydrochloric acid and ammonia were respectively used as the catalyst in the sol (hydrolysis) and the gel (condensation) processes. The PVAF microstructure was then characterized with the scanning electron microscope (SEM), while the effects of the modified PVAF on both mechanical and rheological properties of oil-well cements were examined. Due to the fact that SiO_2_ can be uniformly coated onto the PVAF surface, such modified PVAF can slightly improve the rheology of the cement slurry, while the raw PVAF exhibits poor dispersion at a high dosage. Compared with those of cement stone without PVAF after curing for 28 days at 60 °C, the flexural strength, compressive strength, and elastic modulus of the cement stone incorporated with the modified PVAFs were enhanced by 37.7%, 66.1%, and 50.0%, respectively. The SEM test (EDX) test, XRD test, and thermogravimetric test prove that the SiO_2_ coating on the PVAF surface can promote the hydration of cement clinker and can react with Ca(OH)_2_ to generate CSH gel. The SiO_2_ grafted onto the surface of PVAFs can improve the bond strength at the fiber/cement matrix interface, thus improving the mechanical properties of cement stone.

## 1. Introduction

**Figure 1:**
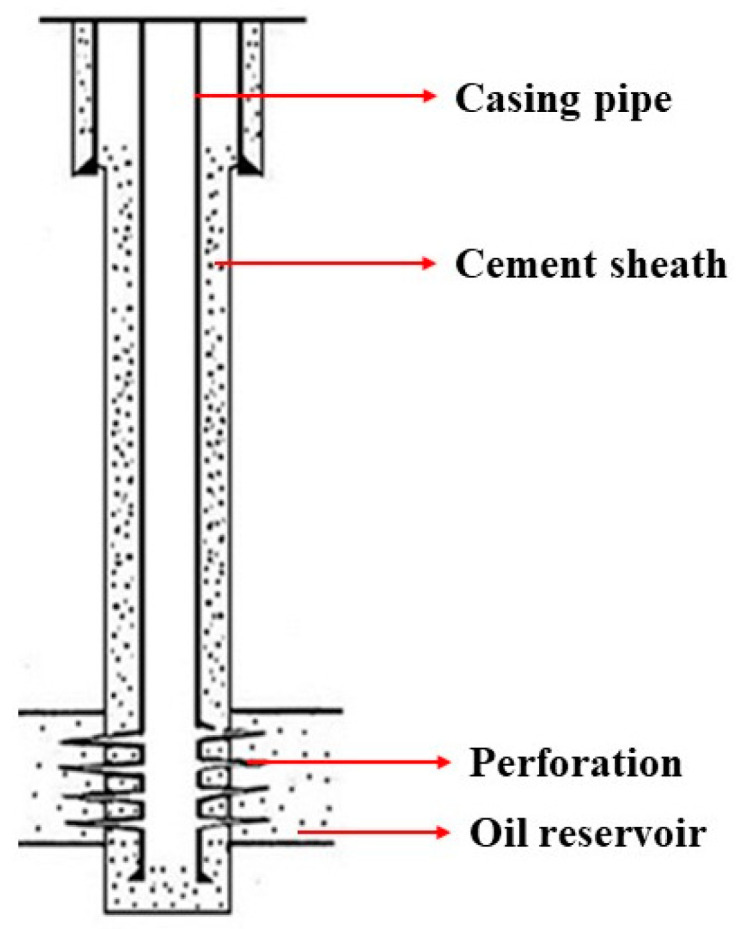
Oil well schematic diagram.

The primary role of the cement sheath of an oil/gas well (see Figure 1, an oil well is a structure where oil is exploited from a deep rock layer) is to isolate the formations (Formation is a rock layer that can store oil or natural gas) from each other as well as support and protect its casing with high quality. Generally, the cement sheath shows the drawbacks of high brittleness, poor tensile strength, and low impact toughness. As for operation and workover in a well, the cement sheath tends to crack owing to complex impact forces, leading to failure of formation isolation, unsustainable casing pressure, other safety issues, and even the abandonment of the well [1,2,3]. The brittle nature of cement-based composite (CBC) makes them vulnerable to loading and impact, subjected to forming cracks. Such an induced crack facilitates the penetration of water and harmful chemicals into the CBC, resulting in dramatic deterioration of the CBC durability [4,5]. As such, it is crucial to enhance the mechanical properties of cement sheath to ensure the efficiency and safety of an oil/gas well.

The introduction of fibers into cement is an essential way to reinforce its mechanic properties and toughness in an oil/gas well. Among the fibers, polyvinyl alcohol fiber (PVAF) has attracted much more attention in the cement industry owing to its high elastic modulus, high acid and alkali resistance, good abrasion resistance, and low cost.

There is still no consensus on the effect of PVAF on the strength of CBC. The majority of the previous studies show that adding PVAF induces a decrease or slight change in the compressive strength of the CBC but an obvious increase in the flexural and tensile strength. Ling et al. [6] found that incorporation of PVAF can greatly enhance the flexural strength (in a range of 5.21–11.17 MPa), tensile strength (1.72–3.44 MPa), and fracture energy of CBC; however, its effect on compressive strength is found to be marginal (varying in a range of 61.8–67.3 MPa) and the PVAF dosage affects the tension zone more than the compression zone. Sun et al. [7] reported that the compressive strength of CBC is reduced with PVAF content, while Niu et al. [8] found that PVAF showed an adverse effect on the compressive strength of sulfoaluminate cement (SAC) with a reinforcing effect on its flexural strength and bending strength. During their testing, a softening performance after undergoing a peak loading was recorded in the SAC owing to the incorporation of PVAF. As for CBC, George et al. [9] found that the addition of 2 vol% PVAF can reduce its compressive strength from 34.69 to 31.69 MPa but enhance its flexural strength from 4.53 to 6.86 MPa.

Other studies, however, found the incorporation of PVAFs can greatly enhance the compressive strength of CBC. By measuring the properties of fly ash together with metakaolin (MK) based geopolymer/alkali-activated mortar incorporated with PVAFs, Zhang et al. [10] found that such incorporated PVAFs improved the compressive strength by 27.6% and fracture performance significantly. By adding 3.0 wt% of PVAFs into an ordinary Portland cement, its compressive strength was found to be enhanced by nearly 40% with a reduction in porosity [11]. Such an increase in comprehensive strength results from the interaction between PVAFs and cement, generating some new compounds that fill the pores and enhance the bond strength at the fiber/cement interface.

The mechanism for improving or reducing the mechanical properties of cement using PVAF or modified PVAF is still unclear. Some researchers demonstrate that the interfacial bond strength at the PVAF/cement matrix interface needs to be enhanced for a higher strength. Zhang et al. [10] stated that a weak bonding strength at the PVAF/cement matrix is the primary challenge for PVAF-reinforced cement owing to insufficient physical and chemical affinity. To address this challenge, SiO_2_ nanoparticles were coated onto PVAFs by using KH560 as a coupling agent. Such modified PVAFs showed a rougher surface and exhibited a higher chemical reactivity with cement. As such, the tensile strength and pullout bonding strength of cement were found to increase up to 21% and 43%, respectively, compared to those of raw PVAFs. Zhang et al. [10] concluded that the reinforcing effect of the modified PVAFs was mainly ascribed to the synergistic effect of friction and chemical bonding forces between SiO_2_ NPs and cement. By modifying the interface between PVAFs and cement matrix by coating graphene oxide on the PVAF, the tensile strength of CBC is found to be enhanced by 35.6% compared to that of CBC mixed with raw PVAFs [12]. No study has been conducted from a perspective of cement hydration reaction to investigate the mechanism for improving the mechanical properties using PVAF and modified PVAF.

This study aims to investigate the effect of coating a SiO_2_ layer onto the PVAF surface to enhance the properties of oil-well cements (OWCs). The properties examined include rheology, compressive, and flexural strengths, while SEM, XRD, and TG analyses were performed to characterize the PVAF and/or OWC to identify the underlying mechanisms.

## 2. Experimental

### 2.1. Materials

The Shengwei Class G cement was purchased from the Shengli Oilfield Huanghe Cementing Technology Service Co., Ltd., SINOPEC., Dongying, Shandong, China). The compositions of the cement can be found in Table 1 [13]. A defoaming agent (Silane type) was purchased from the OMAX Oilfield Technology Co., Ltd., Chengdu, Sichuan, China), while tetraethyl orthosilicate (TEOS), ethanol, hydrochloric acid, and ammonia were provided by the Sinopharm Chemical Reagent Co., Ltd., Shanghai, China). All of these agents are analytically pure. The PVAFs were purchased from the Beijing Kenye Trading Co., Ltd., Bejing, China), and their physical properties are tabulated in Table 2. All the materials used in this study were obtained from the manufacturer.

### 2.2. Modification of PVAFs

#### 2.2.1. Preparation of PVAF Suspension

5.0 g PVAFs and 50 mL water were added into a 100 mL beaker at room temperature; then the system was stirred at a rotation rate of 800 r/min for 1 h (using a constant speed electric mixer) to attain a uniform PVAF suspension.

#### 2.2.2. Preparation of Silica Layer Precursor

5.0 g TEOS, 5.0 g ethanol, and 50 mL deionized water were put into a 100 mL beaker, and the solution pH (pH meter) was adjusted to 2.5 using hydrochloric acid at room temperature. Then, the system was stirred with a rotation speed of 800 r/min until it became homogeneous and transparent, indicating that the hydrolysis of the TEOS was completed and the silica solution as encapsulation precursor was formed. 

#### 2.2.3. Preparation of Modified PVAFs

The silica shell precursor solution was added into the PVAF suspension drop by drop (using a three-necked flask), the pH was adjusted to 9.5 with ammonia, and the system was stirred with a rotation speed of 500 r/min for 0.5 h. After the reaction, the system was cooled to room temperature. Such an obtained product was filtered by suction and washed three times with water, which was subsequently dried in a 60 °C oven until the weight became constant.

### 2.3. Preparation of the Mixed Cement

To prepare the mixed OWCs, specific compositions were selected to examine the effect of PVAFs on the flexural and compressive strengths. The cement without PVAFs was adopted as the control sample and termed COWC, and the OWC samples reinforced with raw PVAFs and SiO_2_-PVAFs were termed as RPVAF-OWC and MPVAF-OWC, respectively. Based on the cement weight, such additives and PVAF contents were calculated. Specifically, the RPVAF-OWC and MPVAF-OWC samples with addition of 0.2 wt%, 0.4 wt%, 0.6 wt%, and 0.8 wt% of PVAFs were denoted as RPVAF-OWC-0.2, RPVAF-OWC-0.4, RPVAF-OWC-0.6, RPVAF-OWC-0.8, MPVAF-OWC-0.2, MPVAF-OWC-0.4, MPVAF-OWC-0.6, and MPVAF-OWC-0.8, respectively. 

Based on the API RP 10B-2 standard [3], the mixing was accordingly performed. Specifically, a dry cement powder and PVAF were mixed together, and water and defoamer were mixed together, respectively, until the two mixtures became uniform. Then, the defoamer solution was poured into the mixing cup, and the dry powder was poured continuously and evenly into the mixing cup with a rotation speed of 4000 r/min within 15 s to mix with the solution. Subsequently, the speed was adjusted to 12,000 r/min, and the stir was kept for 35 s to yield a uniform cement slurry. The proportions of such mixed cement samples are detailed in Table 3.

### 2.4. Characterization of PVAF Surface

The FTIR (ATR-FTIR) spectroscopy was commonly used to characterize the functional groups of PVAFs. In this study, we performed FTIR tests and found that the FTIR spectra of both raw and modified PVAFs show peaks near 1085 and 468 cm^−1^, respectively, indicating that it is difficult to distinguish the two from the FTIR spectra. Thus, we did not include the analysis of FTIR spectra. Instead, we performed the SEM analysis on the surface morphology of the PVAFs using a scanning electron microscope (FEI Nova nano SEM 450, Thermo Fisher, Waltham, MA, USA).

### 2.5. Rheology Tests 

A six-speed rheometer (ZNN-D6, Qingdao HaiTongDa Special Purpose Instrument Co., Ltd., Qingdao, China) was adopted to examine the rheological properties of the OWCs based on the SY/T 6544-2017 standard. The fluidity index *n* and consistency coefficient *K* were respectively calculated with the following equations:

(1a) $$n=2.096\log\left(\frac{\theta_{300}}{\theta_{100}}\right)$$

(1b) $$K=\frac{0.511\theta_{300}}{511^{n}}$$

where $\theta_{100}$ and $\theta_{300}$ are the readings at the shear rate of 100 r/min and 300 r/min, respectively. A larger *n* demonstrates a better slurry fluidity, and a larger *K* indicates a thicker slurry.

### 2.6. Mechanical Properties of OWCs 

After the OWC samples (cubes of 50 mm on each side) have been cured in a high-pressure and high-temperature (HPHT) autoclave for 28 days at 20.7 MPa and 60 °C, the compressive strength tests are performed at a load speed of 1200 ± 100 N/s using the hydraulic machine (WEW-300B, Shandong Jinan Wenteng Test instruments Machinery Co., Ltd., Jinan, China) with a capacity of 200 kN (consistent with the API RP 10B-2 standard). Also, the same apparatus was used to conduct the flexural strength tests for samples with a dimension of 40 mm × 40 mm × 160 mm through a three-point loading configuration at a load speed of 40 ± 5 N/s. To ensure consistent and repeatable measurements, all the compressive and flexural strength results denote the average of three parallel tests.

The elastic modulus was measured based on the GB/T 7897-2008 standard. The measurement equipment adopts a true triaxial test system (RTR-1000, GCTS Company, Tempe, AZ, USA). Samples were examined by using an electro-hydraulic servo universal testing machine (600 kN) (Detroit, MI, USA) by loading them via two phases: For the 1st phase, samples were preloaded and unloaded repeatedly using a displacement loading rate of 1 mm/min, and then the 4th load was initiated as the 2nd phase at the same speed. In addition, the initial load in the 2nd phase was set to be 0.8 kN and kept for 30 s after loading or unloading, while strain was continuously monitored and recorded at the load-holding stage. An equipped data acquisition system is adopted to collect the data generated from the displacement sensor and force sensor, and the sampling frequency is 100 Hz. By definition, the following equation is then applied to determine the elastic modulus,

(2) $$E=\frac{P_{a}-P_{0}}{A\left(\varepsilon_{a}-\varepsilon_{0}\right)}$$

where *E* is the elastic modulus, MPa; *P*_a_ denotes the target load (i.e., 40% of the axial compressive strength); *P*_0_ is the initial load; and *ε*_a_ and *ε*_0_ are the corresponding strains, respectively.

### 2.7. Microstructure Tests of OWCs

Prior to each experiment, the OWC samples were sputter-coated with gold, and then their micro-morphology was evaluated and analyzed through SEM to determine the adhesion between the PVAFs and cement matrix. 

An appropriate amount of sample was taken and stuck onto the conductive adhesive to enter the gold spraying instrument for sample spraying. The gold spraying instrument model is Lycra, EM ACE 200, with current 20 mA for 30 s, and then the sample was placed into the instrument for vacuum testing. The instrument model is an FEI Nova Nano SEM 450 field emission scanning electron microscope from the United States, with a voltage of 20 kV and a current of 200 nA.

### 2.8. XRD Test

The mineralogical structure of cement hydration products was analyzed using an X’Pert PRO MPD X-ray diffractometer (Panalytic Co., Almelo, The Netherlands). Before measurement, the hardened cement sample was ground and passed through a 75 μm IS sieve. Scans were conducted under 30 kV and 10 mA in the range of 5° to 70° (2θ angle) using a rate of 2° per minute and a step size of 0.02°. 

### 2.9. Thermogravimetric Analysis

A thermal analyzer was used for thermogravimetric analysis to study the variation pattern of chemically bound water (non-evaporative water) content in solidified cement paste. The sample preparation method is similar to XRD analysis testing. Approximately 8 mg of the sample was placed into an alumina crucible, and the temperature was raised from room temperature to 105 °C at a rate of 5 °C/min, and 105 °C was maintained for half an hour to remove residual evaporated water. Then, the temperature was raised from 105 °C to 1000 °C at a rate of 5 °C/min. During the experiment, nitrogen gas was used as a protective gas to prevent possible carbonization of the sample.

## 3. Results and Discussion

### 3.1. SEM Images for PVAFs

**Figure 2:**
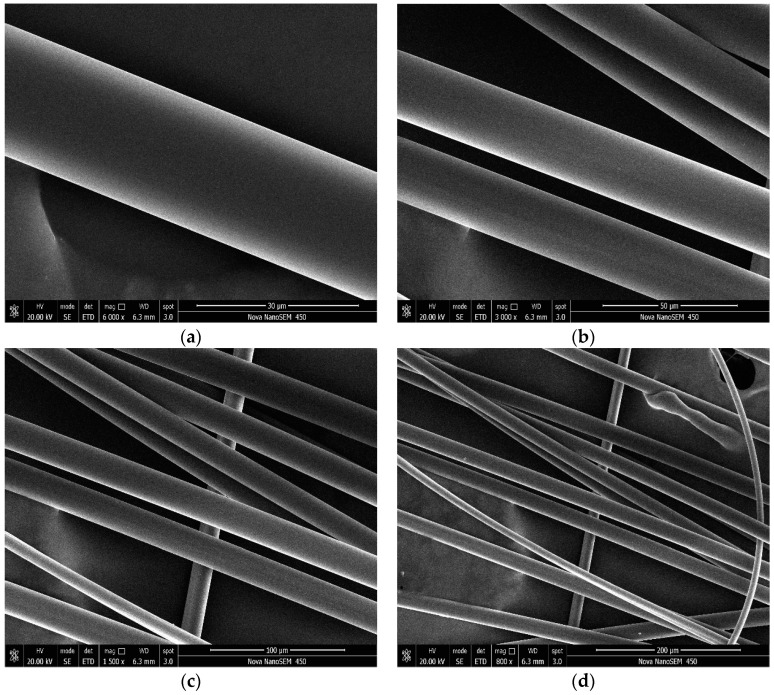
SEM images of raw PVAFs. (**a**) magnify 6000 times; (**b**) magnify3000 times; (**c**) magnify 1500 times; (**d**) magnify 800 times.

**Figure 3:**
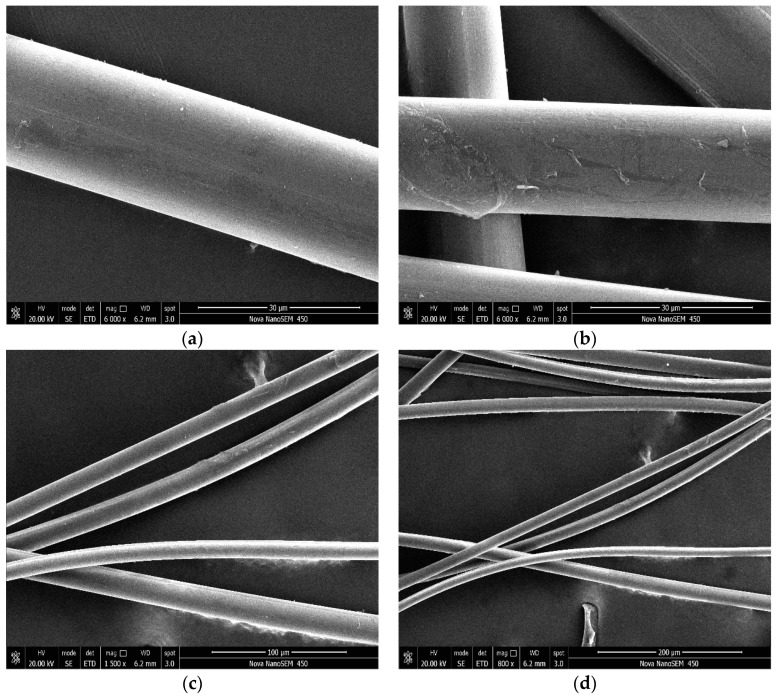
SEM images of the modified PVAFs. (**a**) magnify 6000 times; (**b**) magnify 6000 times; (**c**) magnify 1500 times; (**d**) magnify 800 times.

It can be seen from Figure 2 and Figure 3 that the surface of raw PVAFs is very smooth, while the modified fiber surface shows wrapped outer layers and some scattered bulges, which are mainly the deposited SiO_2_. This indicates that SiO_2_ was successfully coated onto the PVAF surface.

### 3.2. Rheology

As shown in Table 4, the addition of 0.2 wt% of raw PVAFs only slightly changed the rheology of the OWC slurry; however, the OWC samples with additions of 0.4 wt% and 0.6 wt% of raw PVAFs show poor fiber dispersion. Although the ZNN-D6 rheometer showed lower values, the PVAFs tend to agglomerate in these two fiber dosages due to the interactions between fibers (owing to physical and chemical interactions such as hydrogen bonds formed by hydroxyl group). We tried to stir these two samples using a glass rod and found they were very sticky. In this case, the rotational ZNN-D6 rheometer fails to measure the rheological properties of the two OWC samples. This is also related to the structure and working mechanisms of the ZNN-D6 rheometer itself. In comparison, the incorporation of 0.2 wt%–0.6 wt% of the modified PVAF does not show a decreased fiber dispersion, indicating that the coated SiO_2_ on the PVAF surface inhibits the agglomeration of fibers. The OWC samples incorporating the modified PVAFs slightly improve the rheology of the cement slurry. In addition to the shielding effect, the coated SiO_2_ reduces the water adsorption, which may affect the OWC rheology accordingly. 

### 3.3. Mechanical Properties

#### 3.3.1. Compressive Strength

**Figure 4:**
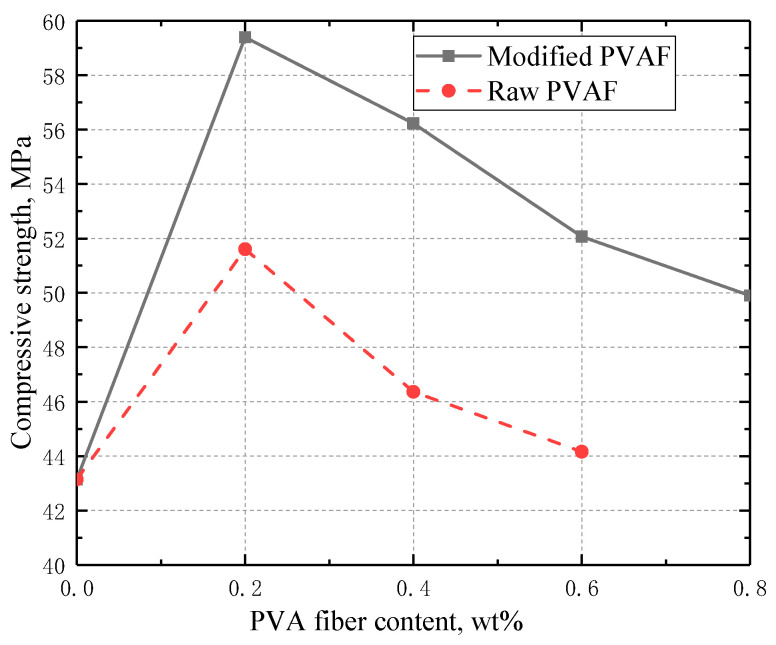
Variation of compressive strength with the fiber content for the two different PVAFs.

As can be seen in Figure 4, the compressive strength of the blank cement sample without PVAFs was measured to be 43.14 Mpa. Incorporation of 0.2 wt% of raw PVAFs can enhance its compressive strength to 51.61 Mpa; however, further increasing the PVAF dosage will reduce the compressive strength. This trend is similar to those observed in the previous studies [14,15]. The reinforcing effect of PVAFs on the compressive strength of CBC is found to depend on both matrix porosity and crack propagation. This is ascribed to the fact that PVAFs can provide a bridging effect to efficiently distribute tensile stresses throughout the crack surface so as to enhance compressive strength and prevent cracks from propagating [16]. Also, the addition of PVAFs increases the matrix porosity due to the agglomerating characteristics of PVAFs, resulting in a decrease in compressive strength [14]. Whether the added PVAFs enhance or reduce the CBC compressive strength depends on the proportion of different effects. The compressive strength of the cement sample with the incorporation of 0.6 wt% of raw PVAFs is only 44.16 Mpa. In comparison, the incorporation of 0.2 wt% of modified PVAFs can increase the compressive strength to 59.4 Mpa. Similar to the samples with raw PVAFs, further increasing the PVAF dosage will reduce the compressive strength; however, the reduction effect is weakened by introducing the modified PVAFs. For example, the compressive strength of the cement samples with the incorporation of 0.4 wt% of modified PVAFs is as high as 56.23 Mpa. The reason for such an improvement in compressive strength with the incorporation of PVAFs is mainly due to a stronger bond strength at the fiber/cement matrix interface [10,12]. Another observed phenomenon is that the raw PVAFs tend to agglomerate at dosages of 0.4 wt% and 0.6 wt%, while the modified PVAFs all dispersed well at different dosages, indicating that the SiO_2_ coated onto the fiber surface inhibits the agglomeration of PVAFs. This is ascribed to the fact that the formation of a hydrogen bond (because of hydroxyl) is inhibited by the coated SiO_2_ layer.

#### 3.3.2. Flexural Strength

**Figure 5:**
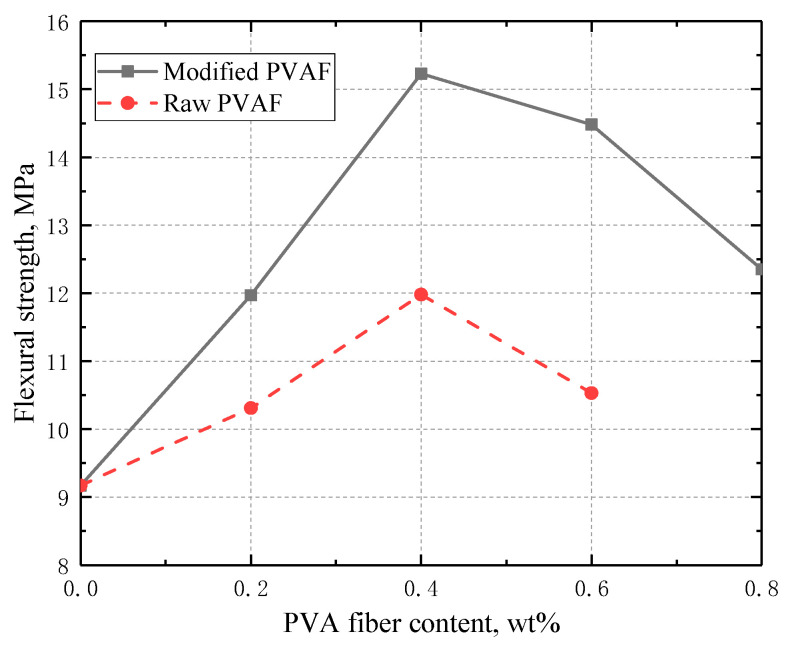
Variation of flexural strength with the fiber content for the two different PVAFs.

The flexural strength of the blank cement sample without PVAF was measured to be 9.17 Mpa (see Figure 5). Incorporation of 0.2 wt% and 0.4 wt% of the raw PVAFs can enhance the flexural strength to 10.31 and 11.98 Mpa, respectively, and further increasing the PVAF dosage will reduce the flexural strength. The flexural strength of the cement sample with the incorporation of 0.6 wt% of the raw PVAFs is 10.53 Mpa. In comparison, the incorporation of 0.2 wt% and 0.4 wt% of modified PVAFs can increase the flexural strength to 12.57 and 15.23 Mpa, respectively. Compared to raw PVAFs, the modified PVAFs impose a much stronger reinforcing effect on the flexural strength. The great enhancements of flexural strength with the incorporation of PVAFs are also observed elsewhere [8,16]. Similar to the samples with raw PVAFs, further increasing the PVAF dosage will reduce the flexural strength; however, the reduction effect is weakened by introducing modified PVAFs. For example, the flexural strength of the cement samples with incorporation of 0.6 wt% and 0.8 wt% of the modified PVAFs is as high as 14.48 Mpa and 12.34 Mpa, respectively. Such a greater improvement of flexural strength with the addition of modified PVAFs results from the enhanced interfacial bond strength and better dispersion compared to those of raw PVAFs.

#### 3.3.3. Elastic Modulus

**Figure 6:**
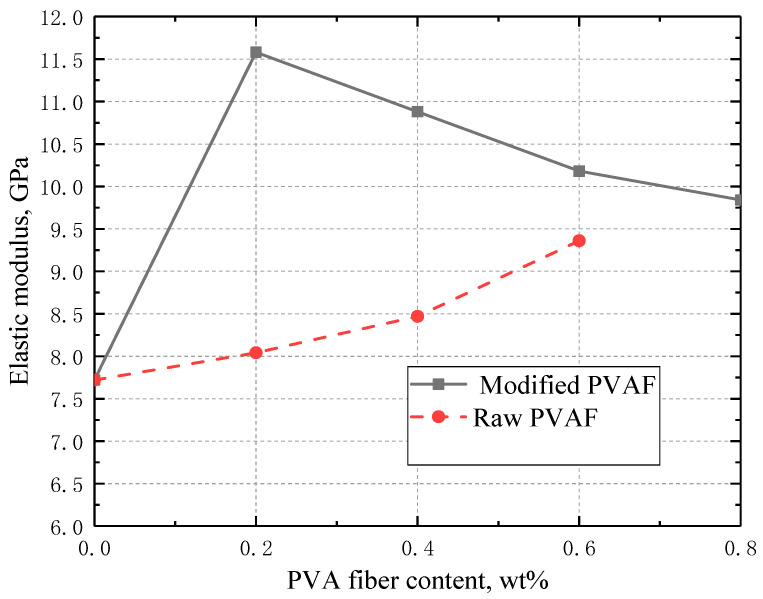
Variation of elastic modulus with PVAF content for the two different PVAFs.

Figure 6 shows the relation of elastic modulus as a function of the PVAF content. The addition of raw PVAFs can moderately improve the elastic modulus, which is increased with the PVAF content. This finding is also documented elsewhere [10]. This is ascribed to the restriction of PVAFs for cement deformation by partially considering the tensile stress used for bearing the axial load. In comparison, the elastic modulus of OWC samples with the addition of modified PVAFs is found to increase first and then decrease with the fiber content. In addition, the reinforcing contribution by incorporating modified PVAFs is much larger than that of the raw PVAFs (e.g., the elastic modulus is enhanced by 50% with an addition of 0.2 wt% modified PVAFs). This is because the interfacial bond strength is improved significantly due to the presence of SiO_2_ on the fiber surface, which enhances the strength of the cement. 

### 3.4. SEM for OWC Micromorphology

**Figure 7:**
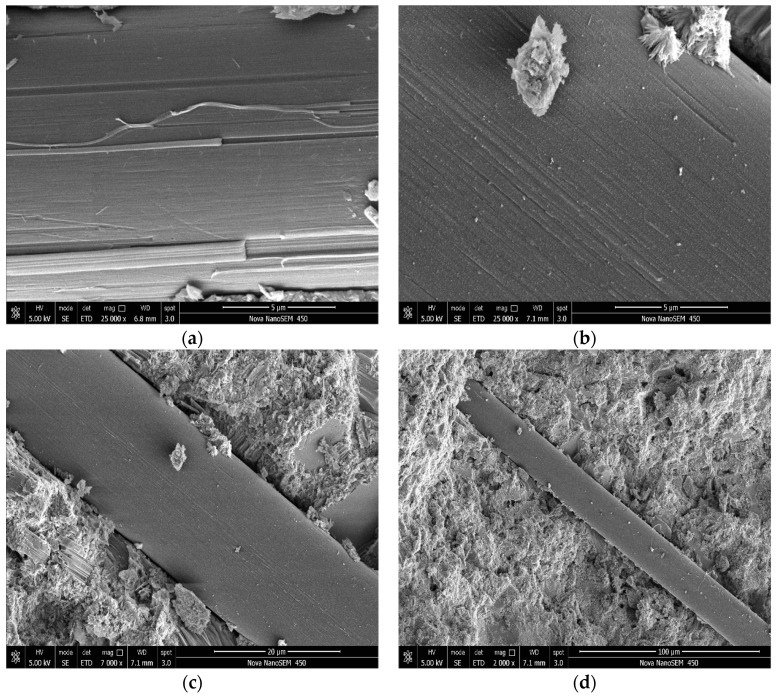
SEM images of OWCs reinforced with raw PVAFs. (**a**) magnify 25,000 times; (**b**) magnify 25,000 times; (**c**) magnify 7000 times; (**d**) magnify 2000 times.

**Figure 8:**
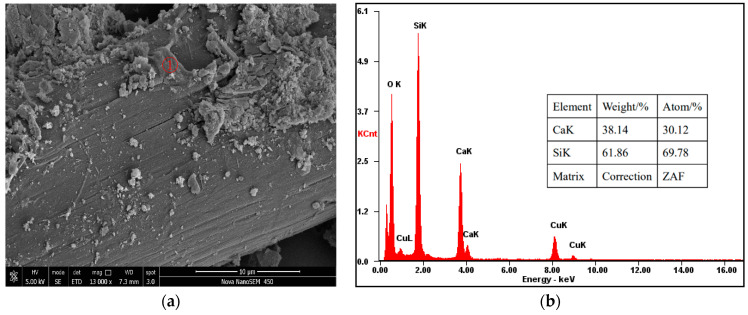
SEM images and EDX of OWC reinforced with the modified PVAFs. (**a**) magnify 13,000 times; (**b**) EDX of the material.

To identify the reinforcing mechanisms for mechanical properties due to the addition of modified PVAFs, the micromorphology of OWC reinforced with the PVAFs was characterized by performing the SEM analysis. It can be seen from Figure 7 that the surface of raw fiber used to reinforce the cement is very smooth, and there is little cement hydration product attached to the fiber surface. In addition, there is a gap between the PVAF and the cement matrix, indicating that the fiber and the cement matrix are not closely connected. This is because the hydrophilic groups of the fiber only form hydrogen bonds or intermolecular forces with the cement matrix but cannot generate chemical bonds. In contrast, as shown in Figure 8a, there are more cement hydration products on the modified PVAF surface used to reinforce cement, and the gap between the PVAF and the cement matrix is not obvious, indicating that the PVAFs and the cement matrix are connected much more closely. This is because the coated SiO_2_ can react with the cement hydration products to form a strong chemical bond, thus effectively increasing the interfacial bonding strength. 

During the SEM test, EDX is used to conduct element analysis on the hydration product. As shown in Figure 8b, the main elements are Ca and Si. From the element composition, it can be confirmed that the product is CSH gel, with a Ca/Si ratio of 0.43. The CSH gel is compact in structure and belongs to type III CSH gel, which significantly improves the interfacial adhesion between PVA fiber and cement [17,18,19].

**Figure 9:**
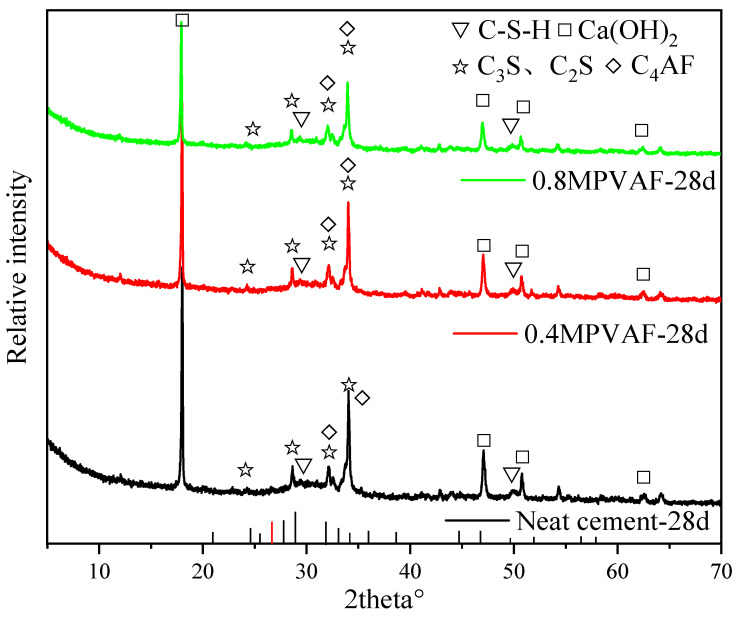
Representative XRD profiles of OWC reinforced with the modified PVAFs.

**Figure 10:**
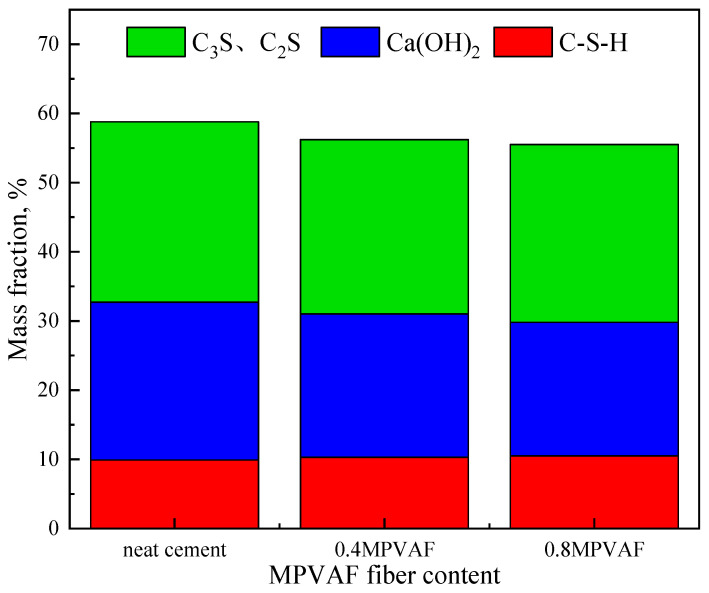
Mineral contents of hydration products of OWC reinforced with the modified PVAFs by QXRD.

After 28 days of curing, the chemical composition of the hydration products of cement stone samples under different MPVAF dosages was analyzed with QXRD (Total Mineral Analysis). It can be seen from Figure 9 and Figure 10 that MPVAF promotes the hydration of cement clinker. With the increase of MPVAF dosage, the acceleration of C_3_S and C_2_S hydration reduces the strength of its characteristic peak and Ca (OH)_2_ peak. Meanwhile, the Ca(OH)_2_ generated by hydration reacts with the deposited SiO_2_ to generate CSH gel, which increases the strength of the C-S-H peak and promotes the formation of more hydrated calcium silicate gel [17,18,19].

**Figure 11:**
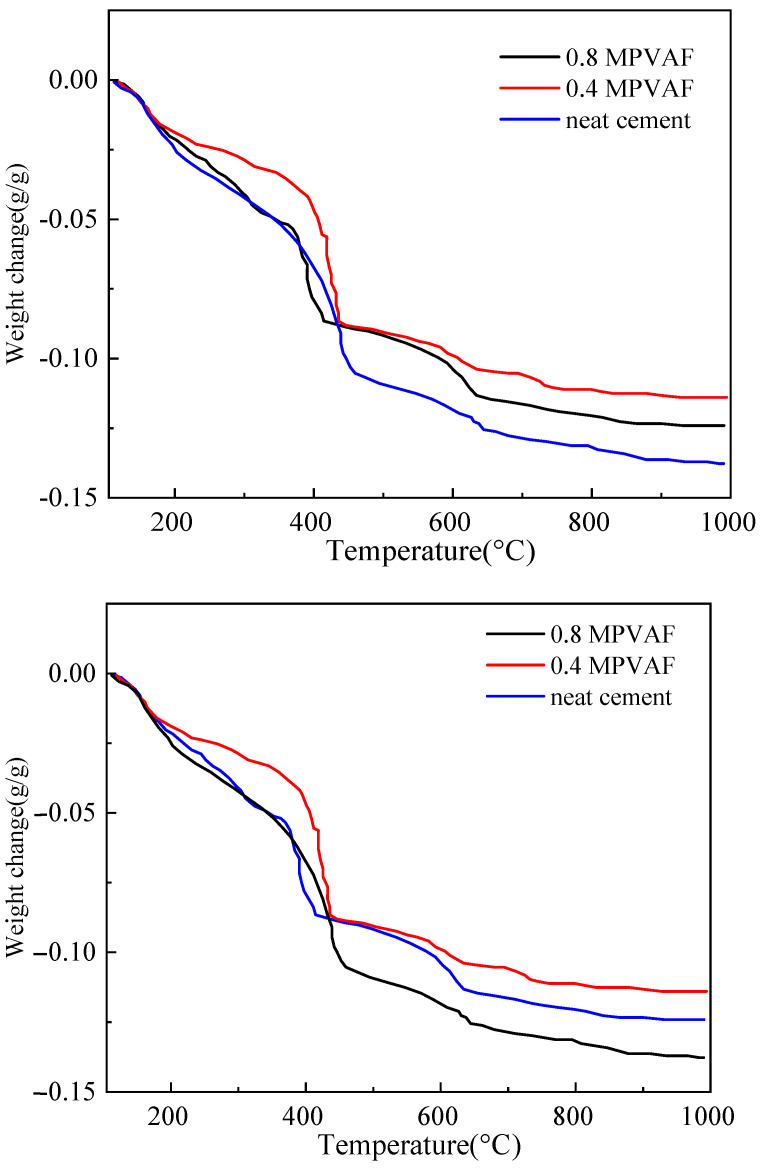
The thermal gravimetry curve of OWC reinforced with the modified PVAFs.

**Figure 12:**
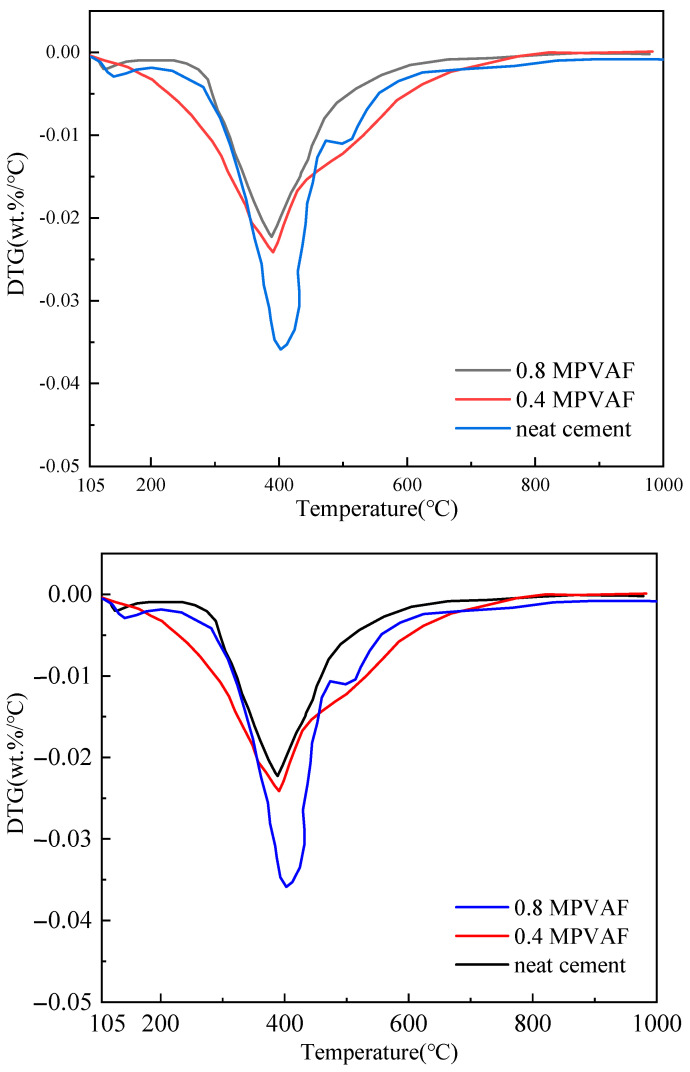
The differential thermal curve of OWC reinforced with the modified PVAFs.

The cement mass loss in the thermogravimetric experiment mainly comes from the thermal decomposition of hydrated calcium silicate gel (C-S-H), ettringite (AFt), and calcium hydroxide (Ca(OH)_2_). The mass loss in the temperature range of 100–210 °C mainly comes from C-S-H gel and Aft, and the decomposition temperature of Ca (OH)_2_ is about 400–500 °C. It can be seen from Figure 11 and Figure 12 that the total weight loss rate is 11–14% under different MPVAF content. With the increase of MPVAF content, the decomposition amount of Ca (OH)_2_ gradually decreases, indicating that the SiO_2_ coating on the material surface can promote the hydration of cement clinker and can react with Ca(OH)_2_ to generate CSH gel. The thermogravimetric test and XRD test have good consistency [17,18,19].

## 4. Conclusions

By grafting SiO_2_ onto the surface of PVAF through a sol-gel process with TEOS as a silicon source, characterization of modified PVAFs was performed, and the effects of the modified PVAFs on the mechanical properties of oil well cement were examined, and relevant mechanism was analyzed. Specific conclusions are given below:

(1) The compressive strength of the cement stone incorporated with 0.2 wt% of the modified PVAFs was enhanced by 37.7% and 15.1% compared with those of cement stone without and with raw PVAFs, respectively. The flexural strength of the cement stone incorporated with 0.4 wt% of the modified PVAFs was enhanced by 66.1% and 27.1%, respectively, compared with those of cement stone without and with raw PVAFs.
(2) Both compressive and flexural strength are first increased and then decreased with the PVAF content. The enhancing effect of modified PVAFs on elastic modulus is much larger than that of raw PVAF, while the elastic modulus is enhanced by 50.0% with the addition of 0.2 wt% modified PVAF.
(3) The SEM test (EDX) test, XRD test, and thermogravimetric test prove that the SiO_2_ coating on the PVAF surface can promote the hydration of cement clinker, and can react with Ca(OH)_2_ to generate CSH gel. The SiO_2_ grafted onto the surface of PVAFs can improve the bond strength at the fiber/cement matrix interface, thus improving the mechanical properties of cement stone.

## Figures and Tables

**Table 1:** Mineral and chemical compositions of the Shengwei Class G cement.

Chemical Composition (wt%)						Mineral Composition (wt%)			
AI_2_O_3_	CaO	Fe_2_O_3_	MgO	SiO_2_	SO_3_	C_2_S	C_3_A	C_3_S	C_4_AF
2.89	65.08	2.63	0.83	24.76	1.25	30.46	2.80	53.70	8.00

**Table 2:** Physical properties of PVAFs.

Length (mm)	Diameter (μm)	Density (g/cm^3^)	Tensile Strength (MPa)	Elastic Modulus (GPa)
6	17	1.3	1600	40

**Table 3:** Proportions of the prepared OWCs.

Formula	Cement, g	PVAF	Deformer	Water, g	W/C Ratio
PC	900	0	1.8 g (0.2 wt%)	396	0.44
OWC-0.2		1.8 g (0.2 wt%)			
OWC-0.4		3.6 g (0.4 wt%)			
OWC-0.6		5.4 g (0.6 wt%)			
OWC-0.8		7.2 g (0.8 wt%)			

Note: We only denote the dosage (e.g., 0.2) of PVAFs and have not distinguished raw PVAFs from the modified PVAFs in the sample number.

**Table 4:** Results of rheology tests from the ZNN-D6 rheometer.

Sample No.	Reading at Different Rotational Speeds (r/min)					Rheology Parameters	
	300	200	100	6	3	*n*	*K*
PC	120	82	73	27	21	0.4524	3.6487
RPVAF-OWC-0.2	130	106	79	22	18	0.4534	3.9293
RPVAF-OWC-0.4	93 ^a^	66 ^a^	49 ^a^	20 ^a^	13 ^a^	N/A	N/A
RPVAF-OWC-0.6	50 ^a^	36 ^a^	31 ^a^	17 ^a^	13 ^a^	N/A	N/A
MPVAF-OWC-0.2	125	102	75	21	16	0.4650	3.5145
MPVAF-OWC-0.4	127	103	76	21	17	0.4674	3.5178
MPVAF-OWC-0.6	129	106	76	22	17	0.4816	3.2694

^a^ The ZNN-D6 rheometer fails to capture the rheology of the cement slurry in these cases.

## Data Availability

The original contributions presented in the study are included in the article, further inquiries can be directed to the corresponding author.

## Nomenclature

CBC	cement-based composite
MPVAF-OWC	modified PVAF reinforced oil-well cement
OWC	oil-well cement
PVA	polyvinyl alcohol
RPVAF-OWC	raw PVAF reinforced oil-well cement
SEM	scanning electron microscope
TEOS	tetraethyl orthosilicate
